# Barriers and facilitators of hepatitis C screening among people who inject drugs: a multi-city, mixed-methods study

**DOI:** 10.1186/1477-7517-11-1

**Published:** 2014-01-14

**Authors:** Joshua A Barocas, Meghan B Brennan, Shawnika J Hull, Scott Stokes, John J Fangman, Ryan P Westergaard

**Affiliations:** 1 Department of Medicine, University of Wisconsin School of Medicine and Public Health, 1685 Highland Ave, UWMFCB 5th floor, Madison, WI 53705, USA; 2 Center for Women’s Health Research, University of Wisconsin - Madison, 700 Regent Street, Suite 301, Madison, WI 53715, USA; 3 William S. Middleton Memorial Veterans Hospital, 2500 Overlook Terrace, Madison, WI 53705, USA; 4 School of Journalism and Mass Communication, University of Wisconsin - Madison, 821 University Ave, 5115 Vilas Hall, Madison, WI 53706, USA; 5 AIDS Resource Center of Wisconsin, 3716 W. Wisconsin Ave, Milwaukee, WI 53208, USA; 6 Department of Medicine, Medical College of Wisconsin, 9200 W Wisconsin Avenue, Milwaukee, WI 53226, USA; 7 Department of Population Health Sciences, University of Wisconsin School of Medicine and Public Health, 707 WARF Building, 610 North Walnut St, Madison, WI 53726, USA

**Keywords:** Hepatitis C, Screening, Injection drug use, Stigma, Health care access

## Abstract

**Background:**

People who inject drugs (PWID) are at high risk of contracting and transmitting and hepatitis C virus (HCV). While accurate screening tests and effective treatment are increasingly available, prior research indicates that many PWID are unaware of their HCV status.

**Methods:**

We examined characteristics associated with HCV screening among 553 PWID utilizing a free, multi-site syringe exchange program (SEP) in 7 cities throughout Wisconsin. All participants completed an 88-item, computerized survey assessing past experiences with HCV testing, HCV transmission risk behaviors, and drug use patterns. A subset of 362 clients responded to a series of open-ended questions eliciting their perceptions of barriers and facilitators to screening for HCV. Transcripts of these responses were analyzed qualitatively using thematic analysis.

**Results:**

Most respondents (88%) reported receiving a HCV test in the past, and most of these (74%) were tested during the preceding 12 months. Despite the availability of free HCV screening at the SEP, fewer than 20% of respondents had ever received a test at a syringe exchange site. Clients were more likely to receive HCV screening in the past year if they had a primary care provider, higher educational attainment, lived in a large metropolitan area, and a prior history of opioid overdose. Themes identified through qualitative analysis suggested important roles of access to medical care and prevention services, and nonjudgmental providers.

**Conclusions:**

Our results suggest that drug-injecting individuals who reside in non-urban settings, who have poor access to primary care, or who have less education may encounter significant barriers to routine HCV screening. Expanded access to primary health care and prevention services, especially in non-urban areas, could address an unmet need for individuals at high risk for HCV.

## Background

Infection with hepatitis C virus (HCV) is the most common cause of end-stage liver disease and the most frequent reason for liver transplantation in the United States [1]. Between 3 and 4 million Americans are chronically infected, many of whom will develop cirrhosis and liver cancer in the coming decades. Because of non-sterile injecting practices, HCV is highly concentrated among people who inject drugs (PWID) [2,3]. The HCV prevalence in a study of young PWID in four large US cities was 35%, ranging from 14% in Chicago to 51% in New York City [4]. Among some cohorts of older PWID, HCV prevalence reportedly exceeds 90% [5,6]. Despite this high prevalence, prior research has shown that many PWID, particularly those younger than 30, are unaware of their status [7,8].

Health care costs associated with HCV infection are substantial and forecasted to rise dramatically over the next decade as “baby boomers,” the birth cohort with the highest HCV prevalence, age into the 7th and 8th decade of life [9]. HCV-infected persons have been estimated to incur twice the annual health care expenses and require hospitalizationat three times the rate of HCV-uninfected individuals, after controlling for age and sex [10].

In May 2011, the U.S. Food and Drug Administration (FDA) approved the first two HCV protease inhibitors for the treatment of chronic HCV infection in combination with standard interferon-based therapy [11]. Availability of direct-acting, antiviral drugs represent a new era in therapeutics when most patients with chronic HCV can be cured using agents for a shorter duration and that have a more favorable side effect profile than prior regimens. The prospect that these advances will translate to population-level declines in HCV disease is currently limited by the fact that 50% to 75% of all HCV-infected individuals in the U.S. are unaware of their serostatus [1]. National initiatives to increase case finding have been proposed, including recommendations for routine screening in health care settings [12]. Many PWID and other high-risk individuals lack insurance, however, and may be systematically underserved by clinic-based approaches [2]. Therefore, community-based approaches are also needed to ensure PWID receive HCV screening.

As PWID are a difficult-to-reach population, little is known about the characteristics of those who are and are not screened for HCV. Understanding facilitators and barriers to HCV screening that are encountered by PWID may help guide the construction of interventions aimed at reducing the burden of unrecognized HCV infection. The objectives of this study were to (1) identify individual characteristics associated with HCV screening among PWID who utilized a free needle-exchange program and (2) identify perceived barriers and facilitators of HCV screening among a convenience sample of PWID in the Midwestern United States.

## Methods

### Study participants

We surveyed PWID utilizing a free, multi-site syringe exchange program (SEP) operating in Southern Wisconsin between June and August 2012. The Lifepoint Needle Exchange operates through office-based locations in the cities of Madison and Milwaukee, and via mobile van units that serve the Milwaukee suburbs, rural communities surrounding Madison, and the cities of Kenosha, Waukesha, Janesville and Beloit. Consecutive individuals who speak and read English, were 18 years or older, and reported a history of injecting drugs were invited to participate. Participants provided verbal informed consent and were paid $10 in cash as compensation for completing the survey. The study protocol was approved by the Institutional Review Board at the University of Wisconsin School of Medicine and Public Health.

### Survey administration

We developed an 88-item questionnaire designed to elicit previous experiences with HCV testing. Survey items assessed demographic characteristics, drug use behaviors (e.g., frequency of injection, sharing needles or equipment, and overdose history), and access to medical care (e.g., emergency room utilization, having a primary care provider). Participants were queried about the frequency of previous HCV testing, the results of past HCV tests, and the locations they had received testing. Multiple-choice and short-answer question items were self-administered by the client, who read the survey and recorded responses using a tablet computer. This allowed respondents to provide information dealing with sensitive subjects such as illicit drug use in a private manner, decreasing the likelihood of socially desirable responding.

A second phase of the assessment was a brief interview consisting of several open-ended questions that evaluated participants’ previous experiences with HCV testing. Development of the brief interview items was guided by the Health Belief Model [13-15] and focused on barriers, facilitators and previous experiences with seeking and receiving testing for HCV. The two question items relevant to the current analysis were (1) “What makes it harder for you to get tested for hepatitis C?” and (2) “What makes it easier for you to get tested for hepatitis C?” Responses were hand-transcribed by the interviewer in real time on the tablet computer. Interviewers were instructed to record participants’ responses verbatim. The text of each response was linked to an anonymous identification number assigned to the participant’s survey responses and saved for subsequent thematic analysis, as described below.

### Quantitative data analysis

Descriptive statistics were used to characterize the study sample with respect to the main variable of interest, which was self-report of receiving HCV screening during the previous 12 months. After excluding respondents who reported already knowing they were HCV-positive, we categorized the study sample in two groups, those who reported having received an HCV test in the past year and those who had not. The latter group includes those who have never tested and whose last HCV test was more than one year prior to the study, because the health behavior of the latter group is inconsistent with HCV testing recommendations.

We compared demographic and behavioral characteristics of the two subsets of respondents using t-tests for continuous variables and chi-squared tests for categorical variables. We used simple logistic regression to generate odds ratios and 95% confidence intervals representing bivariate associations between past-year HCV testing and individual characteristics we hypothesized would be important determinants of testing. An alpha level of 0.05 was assumed to indicate statistical significance. To identify factors independently associated with past-year HCV testing, we used multiple logistic regression models to estimate adjusted odds ratios. Variables with significant bivariate associations and those considered *a priori* to be likely predictors of HCV testing were included in an initial multivariate model. A final model was determined by sequentially eliminating covariates with non-significant *P*-values. Statistical analyses were conducted using STATA Version 11 (Cary, NC).

### Qualitative data analysis

Two investigators (JB and MB) conducted the qualitative analysis using an inductive thematic approach [16,17]. First, investigators independently read all interview transcripts for main themes and subcategories. They then met to develop consensus over a coding scheme used for further analysis. Both investigators independently coded all transcripts line-by-line using the coding scheme and discrepancies were resolved by discussion to reach consensus. Inter-rater reliability was 81%. To explore whether barriers and facilitators are perceived differently by respondents tested for HCV in the past year compared to those who were not, we compared the frequency of specific codes among the two subsets of respondents using chi-squared tests.

## Results and discussion

### Quantitative results

Over the 8-week study period, 862 consecutive syringe exchange clients were invited to participate in the study and 553 eligible PWID (64%) agreed to complete the survey. For the present analysis, we excluded 33 respondents who reported knowing they were HCV-infected and received their diagnosis more than 1 year ago because they would have no reason to be tested in the past 12 months, yielding a final study sample of 520. Most respondents resided in the City of Milwaukee (34.9%) or the Milwaukee suburbs (19.2%). A smaller proportion was recruited from the Madison-based office (19.5%), which serves the City of Madison and surrounding, predominantly rural communities.

Characteristics of the study participants are shown in Table 1, stratified by whether they reported testing in the past year. The median age was 28; most participants were male (69%) and white (83%). The neighborhood of residence was described as “suburban” by 42.7%, “urban” by 40% and “rural” by 15.3% of respondents. Overall, 88% of IDUs indicated they had ever received a HCV test, and 73.8% had done so in the past year. Respondents who had reported HCV testing in the past year were asked to specify the location where they received a HCV test most recently. Of 329 PWID tested in the past year, 64 (19.5%) received their test at the SEP. Nearly one third (32.5%) received testing at a primary care medical clinic, and 34 (10.3%) received testing in a correctional facility. The remaining respondents reported they received testing at other health care and public health venues.

**Table 1:** Characteristics of sample, by receipt of HCV test in the past year (N = 520)

**Characteristics**	**Not tested in past 12 m**	**Tested in past 12 m**	***P*** *
Overall number of subjects	136 (26.2)	384 (73.8)	
Age			
<30 years	62 (28.2)	158 (71.8)	0.37
≥30 years	74 (24.7)	226 (75.3)	
Gender			
Male	98 (27.5)	259 (72.5)	0.32
Female	38 (23.3)	125 (76.7)	
Race			
White	108 (26.4)	301 (73.6)	0.80
Non-white	28 (25.2)	83 (74.8)	
Employment status			
Unemployed	90 (28.3)	228 (71.7)	0.27
Employed – part time	23 (25.6)	67 (74.4)	
Employed – full time	23 (20.5)	89 (79.5)	
Has health insurance			
No	84 (30.4)	192 (69.6)	0.02
Yes	52 (21.3)	192 (78.7)	
Has a primary care provider			
No	88 (31.7)	190 (68.3)	<0.01
Yes	48 (19.8)	194 (76.7)	
Area of residence			
Rural	27 (33.8)	53 (66.2)	0.05
Suburban	64 (28.8)	158 (71.2)	
Urban	44 (21.2)	164 (78.8)	
Milwaukee zip code			
No	80 (33.2)	161 (66.8)	<0.01
Yes	56 (20.1)	223 (79.9)	
Education			
Did not finish HS	31 (40.3)	46 (59.7)	<0.01
GED or high school diploma	67 (31.9)	143 (68.1)	
Some college/technical school	29 (17.2)	140 (82.4)	
Graduated college/technical school	9 (14.1)	55 (85.9)	
Years since initiation of injection drug use			
Less than 2 years	40 (32.5)	83 (67.5)	0.19
2-5 years	50 (23.7)	161 (76.2	
More than 5 years	46 (24.7)	140 (75.3)	
Frequency of injection drug use within the past 6 months			
Once per week or less	17 (34.0)	33 (66.0)	0.24
2-6 times/week	33 (29.5)	79 (70.5)	
Once daily or more	86 (24.3)	268 (75.7)	
Shared works in past 6 months**			
No	59 (26.8)	161 (73.2)	0.77
Yes	77 (25.7)	223 (74.3)	
History of opioid overdose			
No	105 (28.9)	259 (71.2)	0.05
Yes	31 (20.4)	121 (79.6)	
Positive test for HCV			
No	n/a	343	n/a
Yes	n/a	41	

All values are n (%) unless otherwise noted.

*P value from chi-squared of independence between selected covariate and receipt of HCV test in the preceding 12 months.

**Sharing works was defined as any report of using a syringe, cooker/container, or cotton filter after another person had already used it.

Table 2 shows the results of univariate and multivariate logistic regression models measuring the association of past-year HCV testing and selected participant characteristics. Those who reported recent testing were more likely to live in urban or suburban areas, to have health insurance, and to have received some education beyond high school. There were no differences in past-year testing according to age, gender, or race. In the final, adjusted model, having a primary care provider (PCP) was independently associated with past-year HCV testing (adjusted OR 2.0, 95% C.I. 1.3 – 3.0), as was higher educational attainment (adjusted OR 1.9, 95% C.I. 1.4 – 2.5), residence in Milwaukee (adjusted OR 2.3, 95% C.I. 1.5 – 3.5), and lifetime occurrence of opioid overdose (adjusted OR 1.8, 95% C.I. 1.1 – 2.8). Moreover, among those who had a PCP, those attending a medical appointment with a PCP during the six months before the study had nearly three times greater odds of having been tested for HCV (univariate OR 2.9, 95% C.I. 1.3 – 6.4).

**Table 2:** Factors associated with receiving an HCV test during the past year (N = 520)

	**Unadjusted OR (95% CI)**	**Adjusted OR (95% CI)**
Age <30 years	1.2 (0.8 – 1.8)	
Female gender	1.2 (0.8 – 1.9)	
Completed college or technical school	1.9 (1.5 – 2.5)	1.9 (1.4 – 2.5)
Currently employed*	1.3 (0.9 – 2.0)	
Has health insurance	1.6 (1.1 – 2.4)	
Has a primary care provider	1.9 (1.2 – 2.8)	2.0 (1.3 – 3.0)
Urban residence**	1.6 (1.1 – 2.4)	
Milwaukee zip code	2.0 (1.3 – 2.9)	2.3 (1.5 – 3.5)
History of opioid overdose	1.6 (1.0 – 2.5)	1.8 (1.1 – 2.8)
Injecting for 2 or more years	1.5 (1.0 – 2.4)	
Injecting daily during past 6 months***	1.4 (0.9 – 2.1)	
Sharing works in past 6 months^±^	1.1 (0.7 – 1.6)	

*Employed full- or part-time as compared with unemployed.

**Compared to a collapsed single group of rural and suburban.

***Compared to a collapsed single group of people who injected less than daily (i.e. combined once a week and 2–6 times a week.

±Sharing works was defined as any report of using a syringe, cooker/container, or cotton filter after another person had already used it.

### Qualitative results

Of the 553 individuals who agreed to complete the survey, 362 (65% of survey respondents) also responded to the brief interview questions. Of 31 respondents that completed the brief interview who reported having a previous positive test for HCV, 13 had been aware of their positive antibody status for more than 1 year, and were excluded from past-year testing analysis. Barriers and facilitators to past-year testing derived from thematic analysis of these responses are shown in Table 3 and Table 4, respectively. There were few differences in the type and frequency of barriers reported by PWID who were tested in the past year compared to those who were not. The frequency of codes representing facilitators of HCV testing was also similar among respondents in the two groups. Commonly-reported barriers and facilitators, emphasized with illustrative quotations, are described below.

**Table 3:** Barriers to past year HCV testing (N = 349)

	**Tested in past 12 m**	**Not tested in past 12 m**	P*
Overall number of subjects (N = 349)	260 (74.5)	89 (25.5)	
Barriers by code**			
Fear of positive test	27 (9.2)	8 (7.5)	0.58
Perceived risk	3 (1.0)	4 (3.7)	0.07
Stigma associated with HCV and/or IVDU	4 (1.4)	3 (2.8)	0.33
Lab characteristic	13 (4.5)	3 (2.8)	0.46
Lack of access to transportation	48 (16.4)	15 (14)	0.56
Time constraints	31 (10.6)	14 (13.1)	0.49
Lack of knowledge of testing	27 (9.2)	11 (10.3)	0.76
Cost	30 (10.3)	11 (10.3)	1.00
Lack of access to MD/PCP	3 (1.0)	0 (0)	0.30
Not having to take initiative	1 (0.3)	1 (0.9)	0.46
Lack of rapport with provider	0 (0)	2 (1.9)	0.02
Confidentiality	2 (0.7)	1 (0.9)	0.80
Lack of motivation	5 (1.7)	5 (4.7)	0.09
Other	2 (0.7)	1 (0.9)	0.80
No barriers identified	96 (33)	28 (26.2)	0.20

All values are n (%) unless otherwise noted.

*P value from chi-squared of independence between selected barrier and receipt of HCV test in the preceding 12 months.

**Some respondents reported more than one barrier.

**Table 4:** Facilitators to past year HCV testing (N = 349)

	**Tested in past 12 m**	**Not tested in past 12 m**	P*
Overall number of subjects (N = 349**)	260	89	
Facilitators by code**			
Health concerns for self or others	65 (15.4)	12 (12.5)	0.47
Perceived risk	15 (3.6)	3 (3.1)	0.83
Lab characteristic	15 (3.6)	3 (3.1)	0.83
Access to transportation	36 (8.6)	11 (11.5)	0.37
Mobile testing center/SEP	75 (17.8)	13 (13.5)	0.31
Adequate time	9 (2.1)	4 (4.2)	0.25
Knowledge of testing	25 (6.7)	9 (9.4)	0.22
Free testing	85 (20.2)	13 (13.5)	0.13
Access to MD/PCP	28 (6.7)	5 (5.2)	0.60
Not having to take initiative	5 (1.2)	0 (0)	0.28
Rapport with provider	10 (2.4)	2 (2.1)	0.86
Confidentiality	8 (1.9)	1 (1.0)	0.56
Motivation	5 (1.2)	1 (1.0)	0.90
Other	16 (3.8)	8 (8.3)	0.06
Nothing	24 (5.7)	11 (11.5)	0.04

All values are n (%) unless otherwise noted.

*P value from chi-squared of independence between selected facilitator and receipt of HCV test in the preceding 12 months.

**Some respondents reported more than one facilitator.

Based on responses to the interview questions, we observed that many PWID described an internal motivation regarding their own and/or another person’s health that influenced their decision to get tested for HCV. One person who had been tested in the past year stated:

Knowing [my HCV status] is something that I need to do to stay healthy. Knowing that I’ll feel better about myself if the results are good makes it easier to get tested.

Similarly, lack of awareness of one’s HCV status was described as a source of anxiety for some respondents. One who had not been tested in the past year bluntly stated, “Not knowing sucks. It doesn’t feel good when you don’t know if you have it or not.” Many participants described a sense of altruism regarding potential health consequences their drug use may have for significant others and community-at-large, and cited this as motivation to seek HCV testing. One participant who had not recently been tested stated, “Knowing that there’s an epidemic and that it can be passed on [makes it easier to get tested].” Another participant who had been tested said, “if I knew I was positive, then I would take caution to not infect my family.” While such comments may not reflect accurate knowledge of how HCV is transmitted, they demonstrate a role that concern for others may play in the decision to be tested for HCV.

Respondents commonly reported that fear was an important psychological barrier to HCV testing. Simply being “not ready” was a common response and numerous PWID indicated they were “scared of the result.” One recently tested participant remarked, “I worry about Hep C more than HIV. I’m afraid of what the result might be.” One individual not tested in the past year admitted, “I’m in denial. I don’t want to hear that I have it”.

While some were fearful of their result, others perceived their risk of contracting HCV as low despite injecting drugs and, therefore, considered HCV testing unnecessary. Low risk assessments were based on 1) never sharing needles, 2) lack of symptoms, and 3) prior negative test result.

Health care factors played an important role in the decision of many PWID to undergo HCV testing. Several respondents pointed to the accessibility of nonjudgmental care providers (i.e., mobile testing, SEPs, and PCPs) as an important facilitator of HCV screening. Those who found HCV testing “easy” described having regular contact and a positive rapport with their primary care provider. One tested individual explicitly stated this as a facilitator to testing: “I’m extremely honest with and have a very good relationship with my doctor.” Some appreciated having screening offered to them as part of routine health maintenance rather than having to take the initiative to ask for testing. One tested individual answered that “when it’s [HCV testing] offered to me on a regular basis [it makes it easier to get tested].” A nonjudgmental and confidential atmosphere was reported to be a facilitator of HCV testing in both traditional medical clinics and community-based settings. Participants identified community-based organizations such as the SEP, mobile testing, and public health departments as organizations that facilitated HCV screening. One individual tested in the last 3 months stated, “I can come here [SEP] and the staff does it for free and it’s confidential.” Similarly, “having a safe environment where people aren’t going to ‘notice you’, such as here [SEP], where you know that other people are here for the same reason” provided participants with comfort and eased their concerns about testing.

Most participants who discussed their experience at a SEP felt that the program provided a safe environment, which fostered communication and improved feasibility of testing. Few participants reported negative experiences in health care settings as barriers to receiving HCV testing. Stigma associated with both injection drug use as well as HCV infection was a barrier among these participants. Those who identified stigma as a barrier used words such as “shame,” “embarrassment,” and “taboo” to describe their experiences. Negative feelings such as embarrassment or a feeling that one is being judged were perceived obstacles to seeking HCV testing. One participant who had never been tested stated:

People know that most of the time you get tested for Hep C because you’re an IV user. People judge you no matter what your results are. That’s the worst feeling ever.

Participants identified other tangible perceived barriers and facilitators to testing. Independent of past-year testing, lack of transportation, time constraints, lack of knowledge surrounding testing, and cost of the test were identified barriers. Conversely, access to transportation, awareness of testing locations, and availability of free testing were facilitating factors for both groups.

## Discussion

In this cross-sectional survey of PWID in Wisconsin, we found that most respondents had been previously tested for HCV. Those who were tested for HCV in the past year were more likely to have a PCP, to have completed some education beyond high school, and to reside in the city of Milwaukee. Qualitative analysis of interview responses reinforced the important roles of HCV test availability and health care access in general to facilitate regular screening for PWID. The findings from our study may provide insight into individual- and structural-level barriers and facilitators to routine testing for high-risk individuals, and inform future efforts to promote HCV testing among PWID.

Compared with respondents from other Wisconsin cities, residents of Milwaukee had more than twice the odds of receiving HCV testing in the year prior to the study. Numerous factors may account for this disparity: Milwaukee is the largest and most densely populated city in Wisconsin and has a higher burden of communicable diseases such as HIV and sexually transmitted infections than most other areas of the state. Appropriately, prevention services such as the Lifepoint Needle Exchange are more numerous and accessible to Milwaukee residents, and PWID in this area may therefore have greater knowledge of available resources. Additionally, individuals living in cities with a higher burden of drug use and HCV may be more likely to encounter peers who have utilized prevention services in the past, to have medical providers who have greater familiarity with the needs of drug-using patients, and to have easier access to primary care or urgent care centers where testing can be performed. There may be unmet needs for community-based services and a paucity of health care providers with experience caring for PWID in less densely populated areas.

We found quantitative evidence that access to health care is an important determinant of regular HCV screening for PWID. Respondents who reported that they have a primary care provider had twice the odds of receiving a test for HCV in the past year as those without at PCP. Though there was no difference in past-year testing, both groups commonly identified access to healthcare professionals as a facilitator to testing. Previous research has noted that continuity of care with a provider has fostered regular screening and, in some cases, adherence to treatment [18]. Our results suggest that PWID are more apt to receive HCV screening when it is offered as a part of routine care, rather than when it is only available “on-demand,” thereby requiring individuals to take initiative for screening themselves. This is consistent with a recent qualitative study indicating that provider-initiated HCV screening is substantially more successful than self-initiated screening among drug users in New York and San Francisco [19]. The previous study, involving focus groups of drug users recruited in both clinical and non-clinical settings, found that while provider-initiated HCV screening was more successful, there was a perceived lack of settings for self-initiated HCV testing yet an eagerness to have access to voluntary testing. This differed from testing for HIV, which individuals perceived as much more easily accessible and were more likely to seek based on their own initiative. While we cannot determine from our data whether HCV testing in Wisconsin is more commonly provider-initiated or patient-initiated, our findings highlight a potentially important role that PCPs have in screening for HCV. Particularly, PCPs could initiate the discussion by talking about the benefits of testing, providing information regarding voluntary testing locations, and being explicit about the lack of judgment on the part of the practitioner.

Study participants reported a range of beliefs related to HCV testing, many of which are consistent with previous work on HIV and HCV testing [20-23]. Fear of a positive test result played a role in the decision of many respondents who were resistant to testing. Low perceived risk also contributed to past-year testing in some cases. Medical providers and SEP staff can be instrumental in supporting participants’ testing in both of these groups. Staff can allay fears about a positive result by citing new HCV treatments as well as support group if found to be HCV positive. Motivational interviewing techniques providing feedback regarding drug-injecting behaviors and actual HCV risk may be useful to help those with perceived low risk get tested [24].

Stigma was a theme in respondents’ discussion of barriers to HCV testing, which is consistent with previous research [23]. Participants in our study did not frequently express concerns about stigma from medical professionals or needle-exchange staff, as has been reported previously [25-27]. In fact, nearly one-third of those tested in the past year in our study had been tested in primary care clinics. Based on our data, it does not appear that healthcare settings are a major impediment to testing. Rather, in depth analysis of “stigma” statements revealed that those respondents described perceiving a more generalized, societal stigma of HCV as a “junkie disease” [25]. This perception highlights an opportunity for health care providers and community-based organizations to help foster safe and accepting environments for testing. This may include assurances of confidentiality, education campaigns regarding other risk factors for HCV, and improved provider-patient communication.

There are several limitations to our study. Despite having a large sample size and a reasonably high response rate of 64%, the respondents to our survey were a convenience sample, which may not be fully representative of PWID in the communities we targeted. Our study was performed in a single Midwestern state with a mix of rural, suburban, and urban participants. The findings, therefore, may not be generalizable to drug-using communities in other regions. As all participants were clients at a SEP, our study sample may exclude a subset of PWID who do not use prevention services and may have a higher risk of HCV. While we attempted to minimize bias due to socially-desirable responding by having participants privately self-administer most sections of the survey, the responses to the in-person interview questions may have been influenced by participants’ knowledge of the study’s main goal, which was to collect information useful for promoting HCV testing among PWID who have not been tested previously.

In theory, early detection of HCV can facilitate referrals to treatment and may reduce the future burden of morbidity from liver disease and even decrease HCV transmission [28]. In the past, treatment for HCV has not been widely available or affordable to PWID, many of whom lack health insurance and generally have poor access to health services. Linking PWID who test positive for HCV to care and evaluating for treatment may, therefore, be difficult. However, currently evolving health insurance reforms could eventually make HCV treatment available to a growing number of patients. In this setting, strategies to improve detection of asymptomatic HCV infection as part of routine primary care could yield substantial public health benefit. Moreover, some evidence suggests that detection of asymptomatic infection and subsequent education may lead to safer injection practices and reduce frequency of injecting among high-risk PWID, thereby promoting HCV prevention even among those who do not access treatment [29,30].

## Conclusions

Our study suggests that access to medical and preventive health services that are responsive to the needs and vulnerabilities of people who inject drugs are important determinants of HCV testing among PWID. Increasing the proportion of PWID who receive screening for HCV in the future will require expanding access to programs that provide voluntary counseling and testing, and promoting recognition among medical providers that HCV screening is an important part of routine preventive care. Given that a plurality of PWID previously tested for HCV in our study had been tested in clinical settings, increasing access to primary care is an important strategy for detecting previously undiagnosed cases of HCV. For PWID who are not routinely engaged in medical care, SEPs may also be an underutilized resource for HCV screening.

## Abbreviations

PWID: People who inject drugs; HCV: Hepatitis C virus; PCP: Primary care provider; SEP: Syringe exchange program

## Competing interests

None of the authors have financial or non-financial competing interests.

## Authors’ contributions

JB: Participated in the design and conception of the initial project including proposal and IRB submission; participated in data collection; performed data analysis; and drafted the manuscript. MB: Assisted in the qualitative section of data analysis and coding, and participated in multiple edits of the manuscript. SH: Participated in the initial design of the study including question formulation and participated in edits of the manuscript. SS: Participated in organization of data collectors. JF: Participated in the design and conception of the project. RW: Conceived of the study, participated in its design and coordination and helped to draft the manuscript. All authors have approved the final manuscript.
